# Supplementary material for: Identification of photoperiod-induced specific miRNAs in the adrenal glands of Sunite sheep (Ovis aries)
Source: Front Vet Sci. 2022 Jul 22;9:888207. doi: 10.3389/fvets.2022.888207 (PMC9354845; doi:10.3389/fvets.2022.888207)
Supplement: Supplementary file 1 [file Data_Sheet_1.ZIP › Supplementary file/miRDeep2 file.zip!/miRDeep2 file/Know_Structure/SPLP42A2/oar-mir-30c.pdf]

miRBase precursor : oar-mir-30c
Total read count : 89467
oar-miR-30c read count : 89291
remaining reads : 176

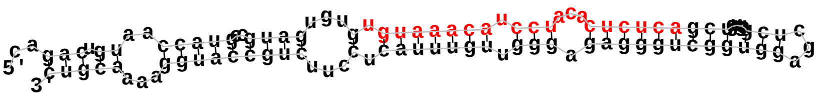

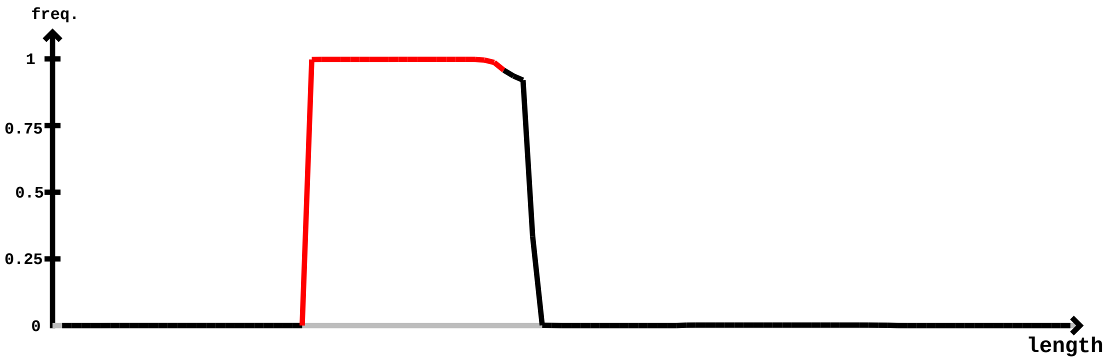


```
                                   oar-miR-30c
5'- cagacuguaaccaugccguagugugcuguaaacauccuacacucucagcugugagcucgagguggcugggagaggguuguuuacuccuucugccauggaaaacguc   -3'    exp
    ..(((.((..(((((..((((...(.(((((((.(((...((((((((....(((...))))))))))).))).))))))).)...)))))))))...)))))    reads    mm    sample
    cagacuguaaccaugccguagugug................................................................................    1        0     seq
    ....cuguaaccaugccguagugug................................................................................    4        0     seq
    .....uguaaccaugccguagugug................................................................................    1        0     seq
    .........................uguCaacauccuacacu...............................................................    1        1     seq
    .........................uguaaacauccuacacA...............................................................    1        1     seq
    .........................uguaaacauccuacacu...............................................................    5        0     seq
    .........................uguaaacauccuacacuc..............................................................    178      0     seq
    .........................uguGaacauccuacacuc..............................................................    1        1     seq
    .........................uguaaacauccuacacuU..............................................................    46       1     seq
    .........................uguaaacaCccuacacuc..............................................................    2        1     seq
    .........................uguaaacaCccuacacucu.............................................................    54       1     seq
    .........................uguGaacauccuacacucu.............................................................    4        1     seq
    .........................uguaaacauccuacacuUu.............................................................    2        1     seq
    .........................uguaaacauccuacacAcu.............................................................    1        1     seq
    .........................uguaaacauccuacacucu.............................................................    728      0     seq
    .........................uguaaacauccuacacucuc............................................................    2421     0     seq
    .........................uguaaacaCccuacacucuc............................................................    175      1     seq
    .........................uguaaacauccuacacucuca...........................................................    1804     0     seq
    .........................uguaaacaCccuacacucuca...........................................................    137      1     seq
    .........................uguaaacaCccuacacucucag..........................................................    100      1     seq
    .........................uguaaacauccuacacucucag..........................................................    1302     0     seq
    .........................uguaaacauccuacacucucagc.........................................................    49030    0     seq
    .........................uguaaacaCccuacacucucagc.........................................................    3308     1     seq
    .........................uguaaacauccuacacucucagcu........................................................    28165    0     seq
    .........................uguaaacaCccuacacucucagcu........................................................    1699     1     seq
    .........................uguaaacaCccuacacucucagcug.......................................................    1        1     seq
    .........................uguaaacauccuacacucucagcug.......................................................    46       0     seq
    .........................uguaaacaCccuacacucucagcugu......................................................    2        1     seq
    .........................uguaaacauccuacacucucagcugu......................................................    41       0     seq
    .........................uguaaacauccuacacucucagcugug.....................................................    1        0     seq
    ..........................guaaacauccuacacucu.............................................................    3        0     seq
    ..........................guaaacaCccuacacucuc............................................................    1        1     seq
    ..........................guaaacauccuacacucuc............................................................    1        0     seq
    ..........................guaaacauccuacacucucagc.........................................................    13       0     seq
    ..........................guaaacaCccuacacucucagcu........................................................    3        1     seq
```

oar-miR-30c

```
cagacuguaaccaugccguagugugUGUAAACAUCCUACACUCUCAgcugugagcucgagguggcugggagaggguuguuuacuccuucugccauggaaaacguc
..........................guaaacauccuacacucucagcu........................................................   10   0   seq
...........................uaaacauccuacacucucag..........................................................   1    0   seq
...........................uaaacauccuacacucucagc.........................................................   1    0   seq
...........................uaaacauccuacacucucagcu........................................................   1    0   seq
............................aaacauccuacacucucagc.........................................................   1    0   seq
............................aaacauccuacacucucagcu........................................................   2    0   seq
..................................ccuacacucucagcugugagcu.................................................   1    0   seq
................................................................cugggagaggguuguuuac......................   3    0   seq
................................................................cugggagaggguuguuuacu.....................   33   0   seq
................................................................cugggagaggguuguuuacuc....................   25   0   seq
................................................................cugggagaggguuguuuacucc...................   76   0   seq
................................................................cugggagaggguuguuuacuccu..................   5    0   seq
.................................................................ugggagaggguuguuuacu.....................   3    0   seq
.................................................................ugggagaggguuguuuacuc....................   4    0   seq
.................................................................ugggagaggguuguuuacucc...................   18   0   seq
.................................................................ugggagaggguuguuuacuccuu.................   1    0   seq
```
